# RanBP2/Nup358 Mediates Sumoylation of STAT1 and Antagonizes Interferon-α-Mediated Antiviral Innate Immunity

**DOI:** 10.3390/ijms25010299

**Published:** 2023-12-25

**Authors:** Jiawei Li, Lili Su, Jing Jiang, Yifan E. Wang, Yingying Ling, Yi Qiu, Huahui Yu, Yucong Huang, Jiangmin Wu, Shan Jiang, Tao Zhang, Alexander F. Palazzo, Qingtang Shen

**Affiliations:** 1 Department of Immunology, School of Basic Medical Sciences, Fujian Medical University, Fuzhou 350108, China; lijiawei_69@163.com (J.L.); su993753394@163.com (L.S.); jiangjing601210@163.com (J.J.); lyy1650820@outlook.com (Y.L.); yuhuahui@fjmu.edu.cn (H.Y.); ychuang2000@163.com (Y.H.); fjmu379664877@outlook.com (J.W.); zy-if-72900yq@outlook.com (S.J.); zjdrzht@fjmu.edu.cn (T.Z.); 2 Department of Biochemistry, University of Toronto, Toronto, ON M5G 1M1, Canada; yifa.wang@mail.utoronto.ca (Y.E.W.); tina.qiu@mail.utoronto.ca (Y.Q.)

**Keywords:** RanBP2, interferon, innate immunity, sumoylation, STAT1, viral infection

## Abstract

Type I interferon (IFN-I)-induced signaling plays a critical role in host antiviral innate immune responses. Despite this, the mechanisms that regulate this signaling pathway have yet to be fully elucidated. The nucleoporin Ran Binding Protein 2 (RanBP2) (also known as Nucleoporin 358 KDa, Nup358) has been implicated in a number of cellular processes, including host innate immune signaling pathways, and is known to influence viral infection. In this study, we documented that RanBP2 mediates the sumoylation of signal transducers and activators of transcription 1 (STAT1) and inhibits IFN-α-induced signaling. Specifically, we found that RanBP2-mediated sumoylation inhibits the interaction of STAT1 and Janus kinase 1 (JAK1), as well as the phosphorylation and nuclear accumulation of STAT1 after IFN-α stimulation, thereby antagonizing the IFN-α-mediated antiviral innate immune signaling pathway and promoting viral infection. Our findings not only provide insights into a novel function of RanBP2 in antiviral innate immunity but may also contribute to the development of new antiviral therapeutic strategies.

## 1. Introduction

Ran Binding Protein 2 (RanBP2), which is also known as Nucleoporin 358 KDa (Nup358), was first identified as a RanGTP binding protein [1,2], that localized to the cytoplasmic filaments of the nuclear pore complex [3]. Aside from the nuclear pore, RanBP2 may also be present in the cytosol, either in annulate lamellae and/or some other biomolecular condensates [4,5,6]. These structures may have mRNAs and interact with other biomolecular condensates including processing bodies (P-bodies) and stress granules (SGs) [4,6,7].

RanBP2 contains an N-terminal tetratricopeptide repeat (TPR) domain, followed by an alpha-helical region, four Ran-binding domains (RBDs 1–4), eight tandem zinc finger motifs, several phenylalanine-glycine (FG) repeats, a small ubiquitin-like modifier (SUMO) E3 ligase domain, and a C-terminal cyclophilin homology domain (CTD) [2,3,8,9]. RanBP2 has been implicated in a number of cellular processes which include nucleocytoplasmic trafficking [10,11,12,13], myogenesis [14,15], trafficking of photoreceptors [16,17], glucose and mRNA metabolism [11,18,19,20], microRNA-induced silencing [21,22], as well as attachment of microtubules to kinetochores during mitosis [23,24,25,26].

The E3 domain of RanBP2 has been shown to form a tight complex at the cytoplasmic filaments of the nuclear pore complex with Ubiquitin Conjugating Enzyme 9 (Ubc9, the only known SUMO E2-conjugating enzyme in humans) and the SUMO-modified RanGAP1 (SUMO-RanGAP1) [27,28,29,30,31,32]. The formation of the RanBP2/SUMO-RanGAP1/Ubc9 complex is essential for the SUMO E3 ligase activity of RanBP2 [33]. Through its SUMO E3 ligase domain, RanBP2 covalently attaches SUMO to distinct substrates such as Ran [34], borealin [35], topoisomerase II [23], and Argonaute proteins [21,22].

RanBP2 has also been reported to modulate the replication of various viruses [36]. In addition, five separate dominant mutations in either the N-terminal or zinc finger region of RanBP2 (T585M, T653I, I656V, T681C, and P1750R) are associated with a pediatric neurological disease called acute necrotizing encephalopathy (ANE1) [37,38,39]. These individuals overproduce cytokines in response to viral infection to generate a “cytokine storm”, which leads to seizures and coma, ultimately resulting in a high rate of mortality, or for survivors, long term neuropathology [37,38,39]. Despite this, the molecular mechanism by which RanBP2 mutations lead to the development of a cytokine storm after viral infection remains unclear.

Previously, we demonstrated that RanBP2 promotes the sumoylation of Argonaute proteins, which enhances microRNA-mediated silencing of two ANE1-associated cytokine mRNAs, Interleukin-6 (IL-6) and tumor necrosis factor-alpha (TNF-α) [22]. Another study revealed that the nucleoporins Nup153 and RanBP2 serve as mediators in the nuclear import of the NF-κB inhibitor (IκBα) upon TNF-α activation, thereby downregulating innate immune responses. Furthermore, when TNF-α-pretreated cells were exposed to Zinc-protoporphyrin IX (ZnPP), a Mallory–Denk bodies (MDBs)-inducing agent, this leads to the accelerated aggregation of IκBα, Nup153, and RanBP2. This prevents the nuclear entry of IκBα and its subsequent binding and termination of NF-κB activation. This novel mechanism may explain why protein aggregates, such as MDBs, induce NF-κB–activation and inflammation during certain liver diseases [40]. Further investigation is needed to determine whether RanBP2 regulates cytokine production through additional means, such as interacting with the antiviral innate immune response after viral infection.

Post-translational modifications (PTMs) play crucial roles in the modulation of protein functions. Small Ubiquitin-like MOdifier (SUMO) is a small protein that is covalently attached to the lysine of target protein substrates. Sumoylation is a dynamic and reversible PTM that can alter target proteins’ localization, stability, and activity [41,42]. The SUMO E3 ligases determine which protein substrates are SUMOylated in a given subcellular region at a given time. Thus far, the main human SUMO E3 ligases include members of the protein inhibitor of activated STAT (PIAS) family, the polycomb protein Pc2, and RanBP2 [41]. Sumoylation has also been shown to engage in the regulation of host immune response to viral infection. In addition, different viruses manipulate the entire process of this cellular machinery to counteract antiviral responses and favor viral replication, propagation and pathogenesis [43,44,45]. Whether the SUMO E3 ligase RanBP2 is involved in the regulation of host antiviral innate immunity remains unclear.

Interestingly, several recent studies have suggested a link between RanBP2 and host immunity. Upon T-cell receptor (TCR) stimulation, protein kinase C-θ (PKC-θ) facilitates RanBP2 subcomplex assembly by phosphorylating RanGAP1, thereby promoting the nuclear translocation of the AP-1 transcription factor in human and murine T-cells [46]. The alpha isoform of the tripartite motif-containing protein 5 (TRIM5α) is a cytoplasmic protein that prevents post-entry retroviral infection, and its antiviral activity is profoundly affected by SUMO [47,48,49]. It has been observed that RanBP2 promotes TRIM5α sumoylation, and loss of RanBP2 dramatically alters the localization of TRIM5α and inhibits its anti-retroviral activity, again illustrating that the importance of RanBP2 in cellular intrinsic antiviral immunity [50].

Type I interferons (IFNs), such as IFN-α and IFN-β, play critical roles in antiviral innate immune responses, which function via autocrine and paracrine signaling to activate Janus kinase (JAK)-signal transducers and activators of transcription (STAT) signaling to induce IFN-stimulated gene (ISG) expression. This is required to repress viral replication, assembly and spreading, as well as to activate immune cells [51,52,53]. Canonical type I IFN signaling initiates with the binding of type I IFNs to IFN-α/β receptor 1 (IFNAR1) and IFNAR2 at the cell surface, which in turn leads to the phosphorylation of JAK1 and tyrosine kinase 2 (TYK2) and subsequent phosphorylation of STAT1 and STAT2 by the activated JAK1 and TYK2. Phosphorylated STAT1 and STAT2 further assemble into a heterodimer that recruits interferon-regulatory factor 9 (IRF9) to form the interferon stimulated gene factor 3 (ISGF3) complex. This complex rapidly translocates into the nucleus to induce the IFN-stimulated response element (ISRE)-driven transcription of ISGs [54]. As a transcription factor, STAT1 is critical for the JAK-STAT signaling pathway. Notably, distinct post-translational modifications modulate the function of STAT1 in the antiviral innate immune response [55,56,57,58,59,60,61,62]. Previous studies have reported that STAT1 is sumoylated at lysine residue 703 by PIAS, and this sumoylation prevents cell hyperresponsiveness to IFN-γ [57,62,63]. However, whether STAT1 is regulated by RanBP2-dependent sumoylation, remains unclear.

In this study, we demonstrate that RanBP2 attenuates interferon-α-mediated JAK-STAT signaling. We observe that RanBP2 mediates STAT1-sumoylation, and inhibits both JAK1-STAT1 interaction and STAT1 phosphorylation induced by IFN-α. This ultimately impedes the nuclear accumulation of STAT1 and ISG activation. In addition, we show that RanBP2 facilitates both herpes simplex virus type 1 (HSV-1) and vesicular stomatitis virus (VSV) infection by negatively regulating type I interferon-mediated antiviral innate immune responses. Our findings suggest that the SUMO E3 ligase RanBP2 is a critical regulator of type I interferon-mediated antiviral innate immunity.

## 2. Results

### 2.1. STAT1 Is a Sumoylation Substrate of RanBP2

**Figure 1:**
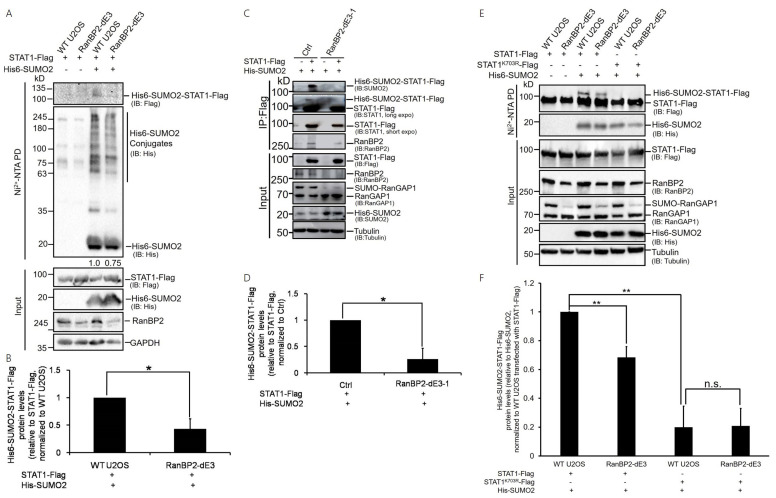
RanBP2 mediates lysine 703 sumoylation of STAT1 in cells. (**A**,**B**) WT U2OS and RanBP2-dE3 cells were co-transfected with plasmids expressing Flag tagged STAT1 (STAT1-Flag) and His-tagged SUMO2 (His6-SUMO2 “+”) or control vector (His6-SUMO2 “−”). Twenty-four hours post-transfection, cells were lysed in 6 M guanidinium chloride, and the His6-SUMO2 conjugates were isolated on nickel beads (“Ni^2+^ NTA PD”) or the lysates were directly analyzed (“input”) and separated by SDS-PAGE. Conjugates were analyzed for the presence of His6-SUMO2-STAT1-Flag by immunoblotting for Flag (IB: Flag), and for total His6-SUMO2 conjugates by immunoblotting for His (IB: His). Input lysates were immunoblotted with antibodies against STAT1-Flag, His6-SUMO2, RanBP2 and GAPDH. Ni^2+^-purified His6-SUMO2 Conjugates and GAPDH protein levels were quantified using densitometric analysis and the ratio of His6-SUMO2 Conjugates/GAPDH (normalized to Ctrl cells) was indicated below relative blots (**A**). The isolated His6-SUMO2-STAT1-Flag and STAT1-Flag (input) protein levels were quantified using densitometric analysis and the ratio of His6-SUMO2-STAT1-Flag/STAT1-Flag was normalized to WT U2OS cells and plotted, with each bar representing the average of two independent experiments ± SEM (**B**). (**C**,**D**) Unmodified (Ctrl) and RanBP2-dE3-1 HEK293 cells were co-transfected with plasmids expressing His6-SUMO2 and STAT1-Flag (lanes 2 and 4), or transfected with plasmids expressing His6-SUMO2 (lanes 1 and 3, negative control). Cell lysates were immunoprecipitated with anti-Flag antibody (IP: Flag) or were directly analyzed (Input) and separated by SDS-PAGE. Samples were analyzed by immunoblotting using different antibodies as indicated, respectively, (**C**). The precipitated His6-SUMO2-STAT1-Flag and STAT1-Flag (input) protein levels were quantified using densitometric analysis and the ratio of His6-SUMO2-STAT1-Flag/STAT1-Flag was normalized to Ctrl cells and plotted, with each bar representing the average of two independent experiments ± SEM (**D**). (**E**) WT U2OS and RanBP2-dE3 cells were co-transfected with plasmids expressing Flag tagged STAT1 (STAT1-Flag) or a lysine 703 to arginine mutant STAT1 (STAT1^K703R^-Flag) together with His-tagged SUMO2 (His6-SUMO2 “+”) or control vector (His6-SUMO2 “−”). Twenty-four hour post-transfection cells were lysed in 6 M guanidinium chloride, and the His6-SUMO2 conjugates were isolated on nickel beads (“Ni^2+^ NTA PD”) or the lysates were directly analyzed (“Input”) and separated by SDS-PAGE. Conjugates were analyzed for the presence of His6-SUMO2-STAT1-Flag by immunoblotting for Flag (IB: Flag), and for His6-SUMO2 by immunoblotting for His (IB: His). Input lysates were immunoblotted with antibodies against STAT1-Flag, RanBP2, RanGAP1, His6-SUMO2, and tubulin. (**F**) The isolated His6-SUMO2-STAT1-Flag and His6-SUMO2 protein levels were quantified using densitometric analysis and the ratio of His6-SUMO2-STAT1-Flag/His6-SUMO2 was normalized to WT U2OS cells transfected with STAT1-Flag and plotted, with each bar representing the average of three independent experiments ± SEM, * *p* < 0.05, ** *p* < 0.01, n.s. indicates no significant difference (Student’s *t*-test). The numbers on the left of each blot indicate the molecular masses of marker proteins in kilodaltons.

To facilitate the initial experiments for investigating the sumoylation substrates of RanBP2, we expressed His-tagged SUMO2 (His6-SUMO2) in unmodified (wild type or “WT”) and RanBP2 dead E3 (“RanBP2-dE3”) human osteosarcoma (U2OS) cell lines, which we generated previously by CRISPR/Cas9 [22]. These cells contain modified forms of RanBP2 that lack SUMO E3 ligase activity. Total His6-SUMO2-conjugated proteins were purified on a nickel column under denaturing conditions, and the eluate was analyzed by anti-His immunoblotting. The level of His6-SUMO2-conjugated proteins in RanBP2-dE3 cells was modestly lower than that of WT U2OS cells (Figure 1A), similar to our previous findings [22]. To validate whether RanBP2 sumoylates STAT1 in vivo, we monitored the sumoylation of exogenously expressed STAT1-Flag. Although total levels of STAT1-Flag were the same, we observed that the level of His6-SUMO2-conjugated STAT1-Flag was lower in RanBP2-dE3 cells in comparison to WT U2OS cells (Figure 1A,B and Figure S1). No STAT1-Flag was seen in the nickel-bound fraction from cells that did not express His6-SUMO2, indicating that the signal was specific to His6-SUMO2-STAT1-Flag.

To further confirm the results, we either co-expressed His6-SUMO2 and STAT1-Flag or expressed His6-SUMO2 alone in the unmodified (Ctrl, transfected with CRISPR/Cas9 empty vector, px459-V2.0) and RanBP2-dE3-1 human embryonic kidney (HEK293) cells previously generated by CRISPR/Cas9 system [22]. Like their U2OS counterparts, the RanBP2-dE3-1 cells have modified forms of RanBP2 that lack SUMO E3 ligase activity. Cell lysates were immunoprecipitated with an anti-Flag antibody, and immunoblotted with an anti-SUMO2, anti-STAT1, or anti-RanBP2 antibody, respectively. The sumoylated form of STAT1 protein was detected via immunoblotting with an anti-STAT1 antibody (Figure 1C), and could be recognized by the anti-SUMO2 antibody in the precipitates from unmodified cells but was barely detected in the precipitates from RanBP2-dE3-1 cells (Figure 1C,D), demonstrating that RanBP2 mediates the sumoylation of STAT1 in these cells. In addition, RanBP2 was present in STAT1-Flag immunoprecipitates in unmodified HEK293 cells and relatively weakly in RanBP2-dE3-1 cells (Figure 1C), which might be due to the lower level of RanBP2 in RanBP2-dE3-1 cells (Figure 1C). Thus, it appears that RanBP2 binds to STAT1 through a region outside of its E3 domain.

To further explore RanBP2 sumoylation sites of STAT1, we analyzed the coding sequence of full-length STAT1 using the SUMOsp2.0 software [64] for potential sumoylation sites, and it predicted lysine 703 as a likely modified residue. We then evaluated whether RanBP2 sumoylated STAT1 at lysine 703 by testing both wildtype STAT1 (STAT1-Flag) and a lysine-to-arginine mutant (STAT1^K703R^-Flag) in the in vivo sumoylation assay, in both WT and RanBP2-dE3 U2OS cells. Again, a lower level of His6-SUMO2-conjugated STAT1-Flag was seen in RanBP2-dE3 cells in comparison to WT U2OS cells (Figure 1E,F). Although total levels of STAT1^K703R^-Flag were the same as STAT1-Flag, the K703R mutation completely abolished STAT1 sumoylation in both WT and RanBP2-dE3 U2OS cells (Figure 1E,F), indicating that lysine 703 of STAT1 is the RanBP2 sumoylation site. Note that a low but measurable sumoylation signal was observed for STAT1-Flag in RanBP2-dE3 cells, suggesting that lysine 703 of STAT1 could also be sumoylated by other SUMO E3 ligases, such as PIAS, as previous studies reported [57,63].

Taken together with our new results, we conclude that STAT1 is sumoylated by RanBP2 at lysine 703 in human cells.

### 2.2. RanBP2 Inhibits the Interferon-α-Mediated Signaling Pathway

**Figure 2:**
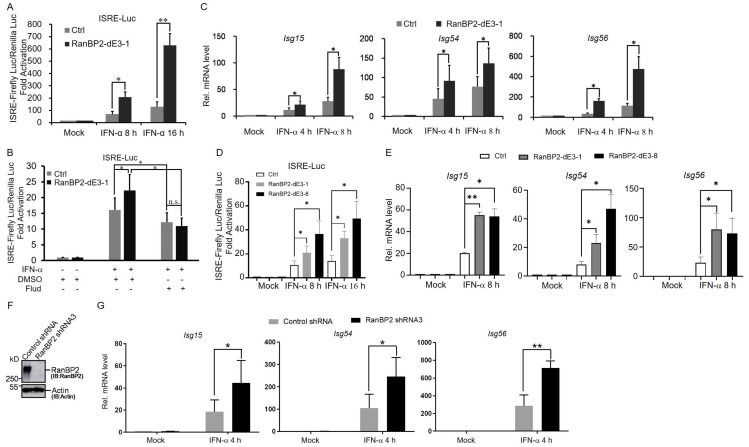
RanBP2 inhibits IFN-α-mediated antiviral innate immunity. (**A**) Unmodified (Ctrl) and RanBP2-dE3-1 cells were co-transfected with 200 ng ISRE-Luc and 50 ng pRL-TK for 24 h, and then mock treated (Mock), or treated with exogenous IFN-α (1000 U/mL) for another 8 h or 16 h and then subjected to dual luciferase assays. Firefly and Renilla luciferase luminescence were measured, and the ratio was normalized to the Ctrl cell line without IFN-α stimulation. Each bar represents the average of three independent experiments ± SD. (**B**) The effect of STAT1 inhibitor fludarabine (Flud) on the activation of ISRE promoters in Ctrl and RanBP2-dE3-1 cells. Unmodified (Ctrl) and RanBP2-dE3-1 cells were co-transfected with 200 ng ISRE-Luc and 50 ng pRL-TK for 24 h, and then mock treated (DMSO) or treated with exogenous IFN-α (1000 U/mL) alone or together with Flud (50 μM) for another 8 h and then subjected to dual luciferase assays. Firefly and Renilla luciferase luminescence were measured, and the ratio was normalized to the Ctrl cell line without IFN-α stimulation. Each bar represents the average of three independent experiments ± SD. (**C**) Ctrl and RanBP2-dE3-1 cells were mock treated (Mock) or treated with exogenous IFN-α (1000 U/mL) for 4 h or 8 h and then subjected to RT-qPCR analysis to detect mRNA levels of *Isg15*, *Isg54* and *Isg56*. The amount of each *Isg* mRNA was normalized to the Ctrl cell line without IFN-α stimulation (Mock). Each bar represents the average of four independent experiments ± SD. (**D**) Unmodified (Ctrl), RanBP2-dE3-1 and RanBP2-dE3-8 cells were co-transfected with 200 ng of ISRE-Luc and 50 ng pRL-TK for 24 h, and then mock treated (Mock) or treated with exogenous IFN-α (1000 U/mL) for another 8 h or 16 h and then subjected to dual luciferase assays. Firefly and Renilla luciferase luminescence were measured, and the ratio was normalized to the Ctrl cell line without IFN-α stimulation. Each bar represents the average of three independent experiments ± SD. (**E**) Ctrl, RanBP2-dE3-1 and RanBP2-dE3-8 cells were mock treated (Mock), or treated with exogenous IFN-α (1000 U/mL) for 8 h and then subjected to RT-qPCR analysis to detect mRNA levels of *Isg15*, *Isg54* and *Isg56*. The amount of each *Isg* mRNA was normalized to the Ctrl cell line without IFN-α stimulation (Mock). Each bar represents the average of three independent experiments ± SD. (**F**) THP-1 cells were infected with lentivirus containing shRNA3 directed against RanBP2, or scrambled shRNA (“control shRNA”). Four days post-infection, cell lysates were collected and analyzed by Western blotting using antibodies against RanBP2 and actin. The numbers on the left of each blot indicate the molecular masses of marker proteins in kilodaltons. (**G**) Control shRNA- and RanBP2 shRNA3-treated THP-1 cells were mock treated (Mock) or treated with exogenous IFN-α (1000 U/mL) for 4 h and then subjected to RT-qPCR analysis to detect mRNA levels of *Isg15*, *Isg54* and *Isg56*. The amount of each *Isg* mRNA in different samples was normalized to the Ctrl cell line without IFN-α stimulation (Mock). Each bar represents the average of three independent experiments ± SD. * *p* < 0.05, ** *p* < 0.01, n.s. indicates no significant difference (Student’s *t*-test).

Because STAT1 is essential for type I interferon-induced signaling and is sumoylated by RanBP2, we next sought to investigate the role of RanBP2 in the type I interferon-mediated signaling pathway. We generated additional RanBP2 sumoylation-deficient mutants in human HEK293 cells, which are commonly used in the study of interferon responses, using CRISPR/Cas9 with a specific guide RNA (“gRNA-dE3-1#”) that we designed previously [22] to target the E3 domain of RanBP2. We obtained several clones, called RanBP2 mut 2 to mut 8 (“mut 1” is the RanBP2-dE3-1 cell line isolated in our previous study), which had almost no detectable SUMO-RanGAP1 compared to the unmodified (Ctrl 1 and 2, transfected with CRISPR/Cas9 empty vector, px459-V2.0) cells (Figure S2A). In many cases these lines also had significantly decreased levels of RanBP2 protein, likely because the modification of the E3 domain destabilizes the protein, as we previously documented [22]. To explore the effect of RanBP2 on the activation of interferon-stimulated response element (ISRE) promoters, a luciferase reporter construct under the control of the ISRE promoter was co-transfected into unmodified and RanBP2 mutant cells with a plasmid expressing an active form of IRF3 (IRF3/5D) that has 5-amino-acid substitutions in its C-terminal domain. This mutant form of IRF3 is constitutively localized to the nucleus where it activates the *IFN-β* promoter directly. The newly produced IFN-β then activates type I interferon signaling. We observed that the IRF3/5D-induced ISRE promoter activity was enhanced in all RanBP2 mutant cells compared to unmodified cells (Figure S2B). Consistent with this, we observed that RanBP2-dE3-1 cells significantly enhanced ISRE promoter activity in response to exogenously added IFN-α (Figure 2A). This was dependent on STAT1, as the increase in ISRE promoter activity was not seen in RanBP2-dE3-1 cells after treatment with fludarabine (Flud) (Figure 2B), an inhibitor of STAT1 signaling [65]. In addition, these mutant cells also displayed an increased expression of ISG mRNAs, including *Isg15*, *Isg54*, and *Isg56*, after stimulation with IFN-α (Figure 2C).

To further validate the roles of RanBP2-dependent sumoylation in regulating the IFN-α-mediated signaling pathway, we repeated these assays with a second RanBP2 mutant HEK293 clone, RanBP2 mut 8 (hereafter referred to as RanBP2-dE3-8) as the protein level of RanBP2 was similar to that of unmodified cells (Figure S2A). When cDNA was amplified and sequenced from this cell line, we could detect a RanBP2 mRNA with the 3 bp deletion from copy f1 and a second mRNA missing exon 21 from copy f2 of the gene (Figure S3A–C). Both the 3 bp deletion (copy f1) and the omission of exon 21, with 171 nucleotides (copy f2), do not alter the reading frame of downstream exons (Figure S3D,E). It is likely that this cell line has higher levels of RanBP2 than the other CRISPR/Cas9-modified lines, because the deletion of one amino acid in copy f1 resulted in minimal change to the overall structure of the encoded protein. Despite this, the two mutations are predicted to disrupt RanBP2’s E3 domain, and subsequently inhibit its interaction with Ubc9. Indeed, we observe that in this cell line, there is a drastic decrease in the interaction between RanBP2 and Ubc9, and no detectable RanGAP1 sumoylation (Figure S3D–F). Consistent with our results with RanBP2-dE3-1 cells, we observed that RanBP2-dE3-8 cells significantly enhanced ISRE promoter activity and increased expression of ISG mRNAs, including *Isg15*, *Isg54*, and *Isg56*, in response to exogenously added IFN-α (Figure 2D,E).

To determine whether RanBP2 regulates ISG expression in immune cell lines, we depleted RanBP2 in human myeloid leukemia mononuclear (THP-1) cells using lentiviral delivered shRNA (“RanBP2 shRNA3”, Figure 2F) and examined the expression of ISG mRNAs induced by IFN-α. In agreement with our other results, we observed that RanBP2-depletion resulted in a significant increase in the mRNA expression of *Isg15*, *Isg54* and *Isg56* when compared to control cells (“Control shRNA”, Figure 2G).

These findings suggest that RanBP2 negatively regulates interferon-α-induced signaling in multiple human cell lines.

### 2.3. RanBP2 Inhibits STAT1 Phosphorylation after IFN-α Stimulation by Impairing the Interaction between STAT1 and JAK1

**Figure 3:**
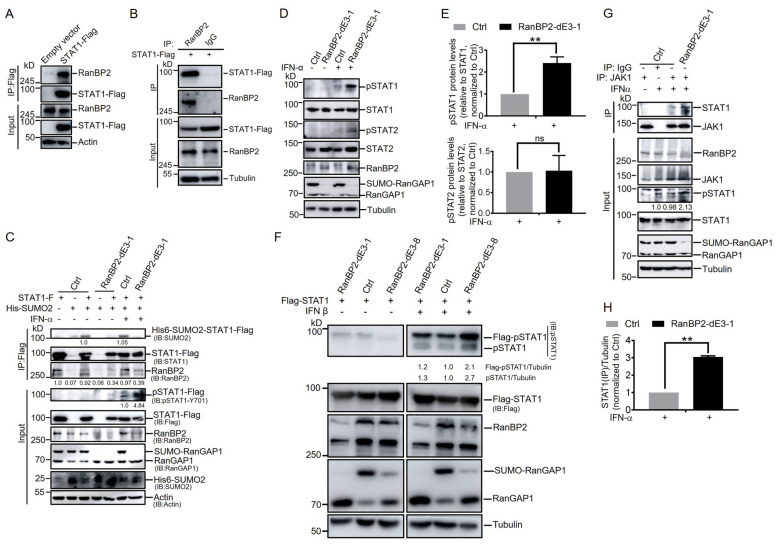
RanBP2 inhibits STAT1 phosphorylation after IFN-α stimulation by impairing the interaction between STAT1 and JAK1. (**A**,**B**) Identification of the interaction between RanBP2 and STAT1 in cells by co-immunoprecipitation (Co-IP). HEK293 cells were transfected with plasmids expressing STAT1-Flag, or empty vector, respectively. Cells were harvested and subjected to Co-IP assays. Cell lysates were separately immunoprecipitated with anti-Flag antibody (**A**) or anti-RanBP2 antibody (with Rabbit IgG as a negative control for IP) (**B**). Samples before (input) and after (IP) immunopurification were analyzed by immunoblot using anti-RanBP2, anti-Flag or anti-actin antibody, respectively. (**C**) The effect of IFN-α stimulation on the sumoylation of STAT1 mediated by RanBP2. Unmodified (Ctrl), and RanBP2-dE3-1 cells were co-transfected with plasmids expressing His6-SUMO2 and STAT1-Flag (lanes 3, 5, 6 and 7), or transfected with plasmids expressing either STAT1-Flag (lane 1) or His6-SUMO2 (lanes 2 and 4) alone as negative controls for 24 h, and cells were treated with 1000 U/mL IFN-α (lanes 6 and 7) for 30 min before harvesting. Cell lysates were immunoprecipitated with anti-Flag antibody (IP: Flag) or were directly analyzed (Input) and separated by SDS-PAGE. Samples were analyzed by immunoblotting using different antibodies as indicated, respectively. The precipitated His6-SUMO2-STAT1-Flag, the co-immunoprecipitated RanBP2 (IP RanBP2), the phosphorylated STAT1-Flag (pSTAT1-Flag), STAT1-Flag (input) and actin protein levels were quantified using densitometric analysis and the ratio of His6-SUMO2-STAT1-Flag/STAT1-Flag, IP RanBP2/actin, or pSTAT1-Flag/STAT1-Flag was indicated below relative blots. (**D**,**E**) Effect of RanBP2 on the phosphorylation of STAT1 induced by IFN-α. Unmodified (Ctrl) and RanBP2-dE3-1 cells were mock treated (lanes 1 and 2) or treated with 1000 U/mL IFN-α (lanes 3 and 4) for 30 min before harvesting. Cell lysates were analyzed by immunoblotting using anti-pSTAT1, anti-STAT1, anti-pSTAT2, anti-STAT2, anti-RanBP2, anti-RanGAP1 and anti-tubulin antibodies, respectively, (**D**). The pSTAT1, STAT1, pSTAT2 and STAT2 protein levels were quantified using densitometric analysis and the ratio of pSTAT1/STAT1, or pSTAT2/STAT2 was normalized to Ctrl cells with IFN-α treatment and plotted, with each bar representing the average of three independent experiments ± SD (**E**). (**F**) Effect of RanBP2 on the phosphorylation of STAT1 induced by IFN-β. Unmodified (Ctrl), RanBP2-dE3-1 and RanBP2-dE3-8 cells overexpressing Flag-STAT1 were mock treated (lanes 1 to 3) or treated with 1000 U/mL IFN-β (lanes 5 to 7) for 30 min before harvesting. Cell lysates were analyzed by immunoblotting using anti-pSTAT1, anti-STAT1, anti-RanBP2, anti-RanGAP1 and anti-tubulin antibodies, respectively. The Flag-pSTAT1, pSTAT1 and STAT1 protein levels were quantified using densitometric analysis and the ratio of Flag-pSTAT1/tubulin or pSTAT1/tubulin was normalized to Ctrl cells with IFN-β treatment and are indicated below relative blots. (**G**,**H**) RanBP2 impairs the interaction between STAT1 and JAK1 induced by IFN-α. Unmodified (Ctrl) and RanBP2-dE3-1 cells were mock treated (lane 1) or treated with 1000 U/mL IFN-α (lanes 2, 3 and 4) for 30 min before harvesting. Cell lysates were separately immunoprecipitated with either mouse anti-JAK1 antibody (lanes 1, 3 and 4), or mouse IgG (lane 2) as a negative control. Samples before (Input) and after (IP) immunopurification were separated by SDS-PAGE and analyzed by immunoblotting using anti-STAT1, anti-JAK1, anti-RanBP2, anti-pSTAT1, anti-RanGAP1 and anti-tubulin antibodies, respectively. pSTAT1 and STAT1 (input) protein levels were quantified using densitometric analysis and the ratio of pSTAT1/STAT1 is indicated below relative blots (**G**). The protein levels of STAT1 (IP) co-immunoprecipitated with JAK1 and Tubulin were quantified using densitometric analysis and the ratio of STAT1 (IP)/Tubulin was normalized to Ctrl cells with IFN-α treatment and plotted, with each bar representing the average of two independent experiments ± SD (**H**). ** *p* < 0.01, ns indicates no significant difference (Student’s *t*-test). The numbers on the left of each blot indicate the molecular masses of marker proteins in kilodaltons.

To determine how RanBP2 antagonizes the interferon-α-mediated signaling pathway, we first verified that RanBP2 interacts with STAT1 in cells by co-immunoprecipitation (co-IP). In agreement with Figure 1C, we found that endogenous RanBP2 was present in STAT1-Flag immunoprecipitates from HEK293 cells (Figure 3A). No RanBP2 was present in anti-Flag immunoprecipitates from cells lacking STAT1-Flag (Figure 3A). We also observed that STAT1-Flag was present in RanBP2 immunoprecipitates but not in control precipitates with rabbit IgG (Figure 3B), confirming the specificity of this interaction.

Given that STAT1 is sumoylated by RanBP2, we next examined whether interferon-α stimulation affected RanBP2-dependent sumoylation of STAT1. We co-expressed STAT1-Flag and His6-SUMO2 in unmodified (Ctrl) and RanBP2-dE3-1 HEK293 cells, and treated cells with IFN-α before harvesting cells for immunoprecipitation and immunoblotting analysis. Again, STAT1 sumoylation was detected in unmodified (Ctrl) cells, but barely detected in RanBP2-dE3-1 cells. IFN-α stimulation did not affect STAT1-Flag sumoylation in either ctrl or RanBP2-dE3-1 cells (Figure 3C). In addition, IFN-α stimulation did not alter the amount of endogenous RanBP2 co-immunoprecipitated with STAT1-Flag in either Ctrl or RanBP2-dE3-1 cells (Figure 3C).

Interestingly, we observed that tyrosine 701 (Y701) phosphorylation of STAT1-Flag (“pSTAT1-Flag”) was significantly enhanced in RanBP2-dE3-1 cells compared to unmodified cells after IFN-α stimulation (Figure 3C). To determine whether this was also true for endogenous STAT1, we treated unmodified and RanBP2-dE3-1 HEK293 cells with IFN-α for 30 min before harvesting and probing for Y701 phosphorylation. Again, the phosphorylation of the endogenous STAT1 was significantly elevated in RanBP2-dE3-1 cells when compared to unmodified cells after IFN-α stimulation (Figure 3D,E). Note that the phosphorylation of the endogenous STAT2 was slightly increased in the RanBP2-dE3-1 cell lines with IFN-α treatment (Figure 3D,E). Despite this, the change in the ratio of pSTAT2/STAT2 was small in comparison to differences in the ratio of pSTAT1/STAT1. The lack of change in the pSTAT2/STAT2 ratio was thus due to an increase in total STAT2 levels in the RanBP2-dE3-1 cell line (Figure 3D,E). We next determined the effect of RanBP2 on the phosphorylation of both Flag-STAT1 and endogenous STAT1 after stimulation with another type I interferon (IFN-β). Once again, the phosphorylation of both Flag-STAT1 and endogenous STAT1 was elevated in RanBP2-dE3-1 cells compared to unmodified cells after IFN-β stimulation (Figure 3F). Consistent with RanBP2-dE3-1 cells, we observed that RanBP2-dE3-8 cells significantly enhanced the phosphorylation of both Flag-STAT1 and endogenous STAT1 in response to exogenously added IFN-β (Figure 3F). To further investigate how RanBP2 might prevent STAT1 phosphorylation, we tested whether RanBP2-dependent sumoylation affected the interaction between STAT1 and its kinase JAK1. We observed that the endogenous JAK1-STAT1 interaction was significantly enhanced in RanBP2-dE3-1 cells compared to unmodified HEK293 cells after IFN-α stimulation (Figure 3G,H). These data suggest that RanBP2 suppresses IFN-α-induced phosphorylation of STAT1 by interfering with the JAK1-STAT1 interaction.

### 2.4. RanBP2 Inhibits the Nuclear Translocation of STAT1 upon IFN-α Stimulation

**Figure 4:**
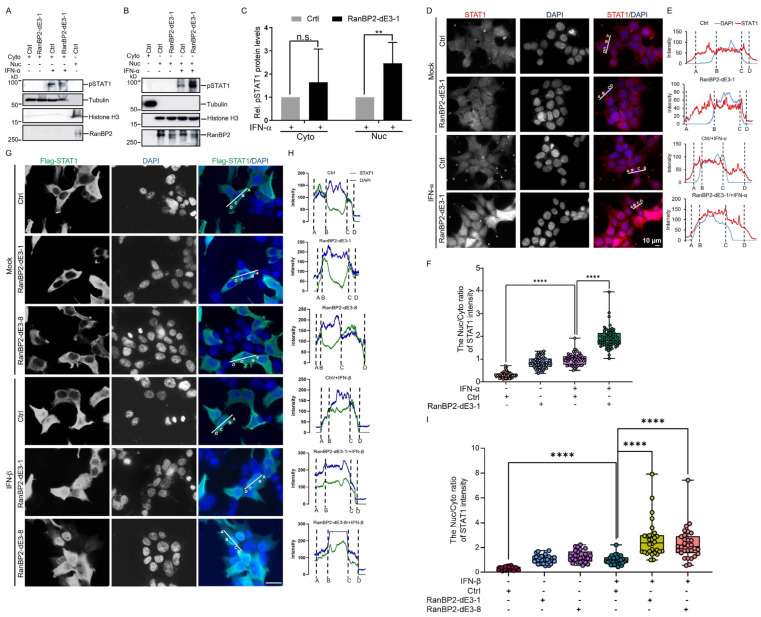
RanBP2 inhibits STAT1 accumulation in the nucleus induced by IFN-α. (**A**,**B**) Unmodified (Ctrl) and RanBP2-dE3-1 HEK293 cells were treated with 1000 U/mL IFN-α for 30 min before harvesting. Cells were fractionated into cytoplasmic (Cyto) (**A**) and nuclear (Nuc) (**B**) fractions. Samples were separated by SDS-PAGE and immunoblotted for pSTAT1, tubulin (cytosolic marker), histone H3 (nuclear marker), and RanBP2. The numbers on the left of each blot indicate the molecular masses of marker proteins in kilodaltons. (**C**) The pSTAT1 protein levels induced by IFN-α in the cytoplasm and the nucleus in (**A**,**B**) were quantified using densitometric analysis and the ratio of cytoplasmic pSTAT1/tubulin or nuclear pSTAT1/histone H3 was normalized to each Ctrl cells with IFN-α treatment and plotted, with each bar representing the average of three independent experiments ± SD. (**D**) Unmodified (Ctrl) and RanBP2-dE3-1 cells were treated with 1000 U/mL IFN-α for 30 min and then fixed and immunostained for STAT1 (red). Nuclear morphology was visualized using 4′,6-diamidino-2-phenylindole (DAPI, blue). The images were obtained by epifluorescence microscopy. STAT1 and DAPI signals are merged in the right panels. Scale bar = 10 μm. (**E**,**F**) Line scan graphs of the representative cells (**E**) and the Nuc/Cyto ratio of STAT1 intensity quantification of line scan graphs for STAT1 (**F**) are also shown. Error bars indicate SD, n > 60 cells from two replicates. (**G**) Unmodified (Ctrl), RanBP2-dE3-1 and RanBP2-dE3-8 cells overexpressing Flag-STAT1 were treated with 1000 U/mL IFN-β for 30 min and then fixed and immunostained for Flag-STAT1 (green). Nuclear morphology was visualized using 4′,6 diamidino 2 phenylindole (DAPI, blue). The images were obtained by epifluorescence microscopy. Flag-STAT1 and DAPI signals are merged in the right panels. Scale bar = 30 μm. (**H**,**I**) Line scan graphs of the representative cells (**H**) and the Nuc/Cyto ratio of Flag-STAT1 intensity quantification of line scan graphs for Flag-STAT1 (**I**) are also shown. Error bars indicate SD, n > 30 cells from two replicates. ** *p* < 0.01, **** *p <* 0.0001, n.s. indicates no significant difference (Student’s *t*-test for (**C**) and Mann–Whitney test for (**F**,**I**)).

To gain further insight into how RanBP2 suppresses interferon-α-mediated signaling, we next sought to examine whether RanBP2-dependent sumoylation affects the nuclear translocation of STAT1 upon interferon stimulation. Unmodified and RanBP2-dE3-1 HEK293 cells were treated with IFN-α for 30 min before harvesting for nuclear and cytoplasmic subcellular fractionation. Cellular localization of the phosphorylated form of STAT1 (pSTAT1) was then detected by immunoblotting. While it was unclear whether two cell lines displayed a difference in the levels of cytoplasmic pSTAT1 after IFN-α stimulation (Figure 4A,C), RanBP2-dE3-1 cells had significantly higher levels of nuclear pSTAT1 when compared to unmodified (Ctrl) cells (Figure 4B,C). Next, we analyzed the cellular distribution of STAT1 by immunofluorescence microscopy. We found that in untreated cells STAT1 was distributed in both the nucleus and cytoplasm, while IFN-α triggered the nuclear translocation of STAT1 in both cell lines. Despite this, the accumulation of nuclear STAT1 in RanBP2-dE3-1 cells was significantly enhanced when compared to unmodified cells (Figure 4D–F). We next determined the effect of RanBP2 on the nuclear translocation of Flag-STAT1 after IFN-β stimulation. We observed that in untreated cells, Flag-STAT1 was mainly distributed in the cytoplasm, while IFN-β triggered the nuclear translocation of Flag-STAT1 in different cell lines (Figure 4G–I). Once again, the accumulation of nuclear Flag-STAT1 was significantly elevated in RanBP2-dE3-1 cells compared to unmodified cells after IFN-β stimulation. Consistent with RanBP2-dE3-1 cells, we observed that RanBP2-dE3-8 cells significantly enhanced the accumulation of nuclear STAT1, in response to exogenously added IFN-β (Figure 4G–I). Thus, these results indicated that RanBP2 impairs the nuclear translocation of STAT1 after IFN-I stimulation, likely via its SUMO E3 ligase activity.

### 2.5. RanBP2 Promotes Viral Infection by Antagonizing Interferon-α-Mediated Antiviral Innate Immunity

**Figure 5:**
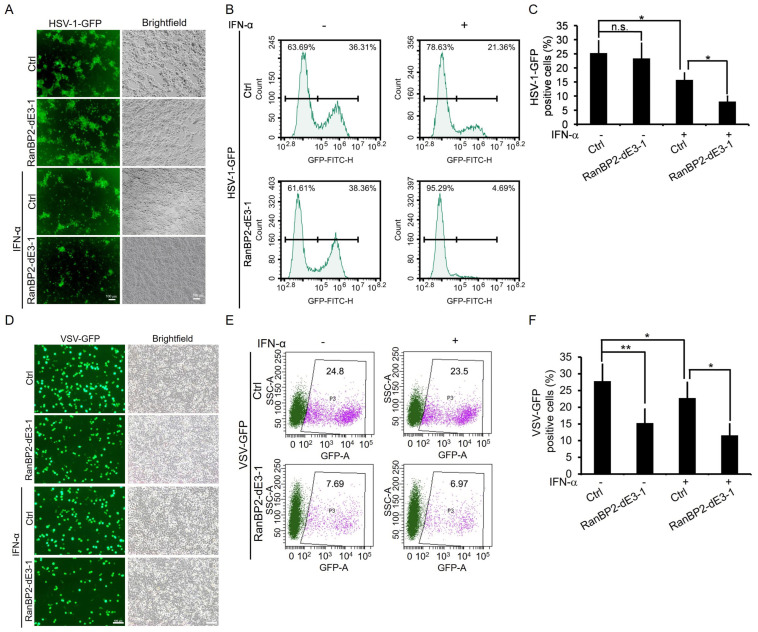
RanBP2 promotes viral infection by impairing IFN-α-induced antiviral innate immunity. Unmodified (Ctrl) and RanBP2-dE3-1 HEK293 cells were treated with 1000 U/mL IFN-α or with medium only (Mock) for 8 h, and then infected with HSV-1-GFP (**A**–**C**) or VSV-GFP (**D**–**F**) at an MOI of 0.2 for 16 h. The infected cells were observed for GFP expression as an indication of HSV-1 and VSV infection. Viral infection was analyzed by fluorescence microscopy (**A**,**D**) and flow cytometry (**B**,**C**,**E**,**F**). Representative images of virus infected (GFP-positive) cells were obtained by epifluorescence microscopy (**A**,**D**). The percentage of virus infected (GFP-positive) cells in (**A**,**D**) were quantified by flow cytometry (**B**,**E**) and plotted (**C**,**F**) with each bar representing the average of three independent experiments ± SEM. Gated regions (GFP-negative and GFP-positive) are indicated by black bars (**B**). Scale bar = 100 μm, * *p* < 0.05, ** *p* < 0.01, n.s. indicates no significant difference (Student’s *t*-test).

Since type I interferon-mediated signaling is crucial for host defense against viruses, we further explored the roles of RanBP2 in host antiviral activity. Unmodified and RanBP2-dE3-1 HEK293 cells were treated with IFN-α before being infected with a DNA virus, Herpes simplex virus type 1 tagged with green fluorescent protein (HSV-1-GFP). Viral infection was measured by fluorescence microscopy and flow cytometry. Note that HSV-1 infection in HEK293 cells, which do not express cyclic GMP–AMP synthase (cGAS) and stimulator of IFN genes (STING) [66,67], does not induce type I interferon, and does not activate IFN-I-mediated antiviral innate immunity. As a result, the number of HSV-1 infected (GFP-positive) cells was comparable between Ctrl and RanBP2-dE3-1 cells without IFN-α treatment. Unsurprisingly, IFN-α treatment decreased the number of GFP-positive cells in unmodified cells. However, the number of GFP-positive cells was significantly lower in RanBP2-dE3-1 compared to unmodified cells after IFN-α stimulation (Figure 5A–C), indicating the critical role of RanBP2-dependent sumoylation in IFN-α-mediated antiviral response.

To further verify whether RanBP2 affects HSV-1 infection by other mechanisms aside from regulating the antiviral innate immunity, we also generated RanBP2 mutant cells (RanBP2-dE3-3) in Vero cell lines, which are incapable of inducing type I interferon upon viral infection [68,69]. As shown in Figure S4A, the protein level of both RanBP2 and SUMO-RanGAP1 were decreased in RanBP2-dE3-3. We then infected Ctrl Vero and RanBP2-dE3-3 cells with HSV-1-GFP. We observed that the efficiency of HSV-1-GFP infection was comparable between Ctrl and RanBP2-dE3-3 Vero cells (Figure S4B–D), confirming that RanBP2 might only affect viral infection by interfering with antiviral innate immunity.

In order to confirm the function of RanBP2 in innate immunity, we then examined whether RanBP2 could regulate the innate defense against an RNA virus, Vesicular stomatitis virus (VSV). We also treated unmodified and RanBP2-dE3-1 HEK293 cells with IFN-α before infecting with VSV tagged with green fluorescent protein (VSV-GFP). Note that VSV infection can induce interferon production by triggering the RIG-I–MAVS signaling pathway [70] and further activate IFN signaling in HEK293 cells. We observed that VSV-GFP infection was significantly inhibited in RanBP2-dE3-1 cells compared to Ctrl cells even without exogenous IFN-α treatment (Figure 5D–F). Consistent with what we observed with HSV-1, exogenous IFN-α stimulation still suppressed VSV-GFP infection in both Ctrl and RanBP2-dE3-1 cells, and the efficiency of VSV-GFP infection was significantly lower in RanBP2-dE3-1 cells compared to Ctrl cells after exogenous IFN-α stimulation (Figure 5D–F).

Taken together, these results suggest that RanBP2-dependent sumoylation dampens interferon-α-mediated signaling and the innate immune response against different types of viruses.

## 3. Discussion

**Figure 6:**
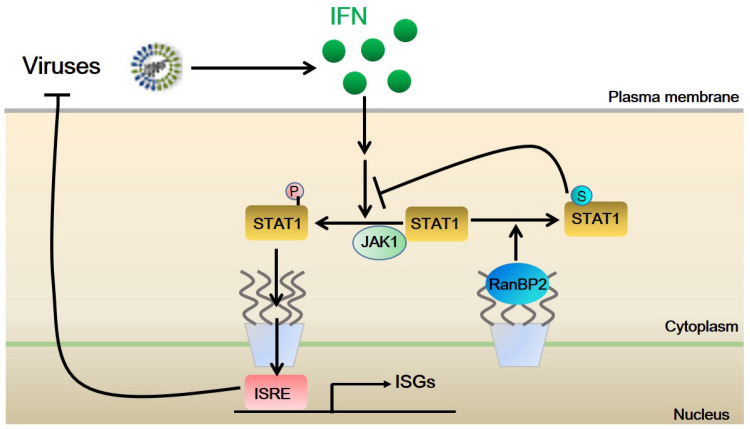
Model for the molecular mechanism of how RanBP2 antagonizes IFN-I-mediated antiviral innate immunity by sumoylating STAT1. P, phosphorylation; S, sumoylation. Arrows represent activation and T lines represent repression.

The function of the SUMO E3 ligase RanBP2 in antiviral innate immunity remains poorly understood. Our recent work suggests that RanBP2 promotes the sumoylation of Argonaute proteins, thereby enforcing microRNA-dependent silencing of proinflammatory cytokines, such as IL-6 and TNF-α [22]. In this study, we identified STAT1 as a sumoylation substrate of RanBP2. Importantly, we found that RanBP2-dependent sumoylation impedes the IFN-α-induced interaction between JAK1 and STAT1 thereby inhibiting STAT1 phosphorylation and nuclear accumulation. We also demonstrated that RanBP2 facilitates both DNA virus (HSV-1) and RNA virus (VSV) infection, by suppressing interferon-α-mediated antiviral signaling likely via its SUMO E3 ligase activity (Figure 6).

It has been reported that RanBP2 interacts with various viruses and affects either viral replication or infection through a variety of different mechanisms [36]. Despite this, previous studies on the interaction between RanBP2 and distinct viruses mainly focused on the effect of RanBP2 on the nucleocytoplasmic trafficking of viral genomes or replication-associated proteins. In this study, we show for the first time, to our knowledge, that RanBP2 is an antagonist for interferon-α-mediated antiviral innate immunity and facilitates both HSV-1 and VSV infection by attenuating the antiviral innate immune responses, likely via its SUMO E3 ligase activity. Whether this is true for other viral infections requires further investigation.

Previous studies suggested that sumoylation of STAT1 on lysine 703 by PIAS negatively regulates IFN-γ-triggered STAT1 activation [57,58,59,60,61,62]. Here, our data suggests that RanBP2 also mediates STAT1 sumoylation, and inhibits IFN-α-triggered activation of STAT1 by reducing its phosphorylation and subsequent nuclear translocation. It is likely that multiple E3 ligases may act on STAT1 to modulate the strength of the innate immune response. This modulation may be disrupted by ANE1 mutations in RanBP2, resulting in cytokine storms upon viral infections. Interestingly, we found that IFN-α stimulation did not alter the sumoylation of STAT1 and that the interaction of STAT1 and RanBP2 occurred in both unmodified and RanBP2-dE3-1 cells (Figure 3C). These results suggest that the phosphorylation of STAT1 induced by IFN-α does not affect sumoylation of STAT1 by RanBP2, and that RanBP2-dependent sumoylation of STAT1 occurs prior to STAT1 phosphorylation. It should be noted that, STAT1 sumoylation was variable in the RanBP2 mutant cells, with it being barely detected in some experiments (Figure 1A–D and Figure 3C) and suppressed but detectable in others (Figure 1E,F). Previous studies have observed PIAS mediates STAT1 sumoylation [57,63] and this may explain some of the variability in our experiments. It would be interesting to determine whether RanBP2 affects the recruitment of PIAS to STAT1, or if it modulates PIAS SUMO E3 ligase activity. RanBP2 has also been implicated in the process of nucleocytoplasmic trafficking by interacting with distinct nuclear transport receptors, such as importin-α, importin-β and exportin-1 [10,11,12,71]. Additional work is needed to determine whether the SUMO E3 ligase activity of RanBP2 interferes with the nuclear accumulation of STAT1 by directly affecting the nucleocytoplasmic shuttling process of phosphorylated STAT1 beyond impairing the phosphorylation of STAT1. Aside from STAT1, the nucleocytoplasmic trafficking of other key innate immunity signal transducers, such as IRF3 and NF-κB (p50/p65), is also critical for the activation of host innate immune response upon viral infection [72]. Here, we observed that VSV-GFP infection was significantly attenuated in RanBP2-dE3-1 cells compared to unmodified cells even without exogenous IFN-α treatment (Figure 5D–F). It should be noted that infection by the RNA virus VSV, but not the DNA virus HSV-1, induces interferon production by activating the RIG-I–MAVS signaling axis [70] and further triggers IFN signaling in HEK293 cells. Therefore, we cannot exclude the possibility that RanBP2 affects VSV infection by other mechanisms, such as regulating the nucleocytoplasmic trafficking of either VSV genomes or VSV replication-associated proteins, or by interfering with the RIG-I–MAVS-IRF3/NF-κB signaling axis. Interestingly, it has been observed that the loss of RanBP2 in mouse motoneurons results in multiple physiological disturbances including profound disruption of Cxcl14/Cxcl12-Cxcr4-Stat3-mediated chemokine signaling, which causes an amyotrophic lateral sclerosis (ALS)-like syndromes with hindlimb paralysis, respiratory distress and premature death [73]. RanBP2-dependent sumoylation also regulates TRIM5α, an anti-viral protein [50]. These observations suggest that RanBP2 likely regulates many innate immune pathways. It will be important to investigate whether RanBP2 modulates additional innate immune signaling pathways by interacting with the signal transducers that are described above.

In conclusion, our study reveals that STAT1 is a sumoylation substrate of RanBP2, and that RanBP2 facilitates viral infection by attenuating interferon-α-induced antiviral innate immunity, likely by sumoylating STAT1. Although our finding sheds new light on a novel function of RanBP2 in antiviral innate immunity, several points should be noted. First, all the experiments in the study were conducted in cultured cells. However, the antagonizing properties of RanBP2 in innate immunity may differ between in vivo and in vitro settings. Second, the role of RanBP2 in antiviral innate immunity may vary depending on different viral infections or various stages of viral infection. Third, determining the stoichiometry of STAT1 sumoylation and phosphorylation, with or without viral infection, will provide insights into the mechanisms underlying the interactions between these two post-translational modifications and their modulation of STAT1 functions in antiviral innate immunity.

## 4. Materials and Methods

### 4.1. Cell Culture, Cell Transfection and Reagents

Human osteosarcoma (U2OS), embryonic kidney 293 (HEK293) and monkey Vero (kindly provided by Yu Chen, Wuhan University, China) cells were maintained in Dulbeco’s Modified Eagle Medium (DMEM) (HyClone, Logan, UT, USA) supplemented with 10% fetal bovine serum (FBS) (PAN, Aidenbach, Germany), and 1% penicillin–streptomycin (BI, Kibbutz Beit-Haemek, Israel). Human myeloid leukemia mononuclear (THP-1) cells (obtained from the American Type Culture Collection) were cultured in RPMI (HyClone, Logan, UT, USA) with 10% FBS and 1% penicillin–streptomycin. All cells were cultured at 37 °C in a 5% CO_2_-humidified incubator. Transient transfections of plasmids were performed with JetPRIME (Polyplus, Illkirch-Graffenstaden, France) for U2OS cells, or Lipofectamine 2000 (Invitrogen, Carlsbad, CA, USA) for HEK293 cells according to the manufacturers’ protocols. For chemical treatments, recombinant human IFN-α (purchased from Cyagen, Santa Clara, CA, USA) were dissolved in sterile distilled water or aqueous buffer containing 0.1% BSA and used at a final concentration of 1000 U/mL. The protease inhibitor mixture cocktail and phosphatase inhibitor cocktail were purchased from AbMole (Shanghai, China), and N-ethylmaleimide (NEM) was purchased from Sigma Aldrich, St. Louis, MO, USA.

### 4.2. Plasmid Constructs

The human His6-SUMO2 (in pcDNA3) and V5-Ubc9 plasmids were gifts from L. Frappier [74,75]. The pCDNA3.1 plasmid containing STAT1-Flag was a gift from X. Cao [55]. The CRISPR/CAS9 plasmids, pSpCas9(BB)-2A-Puro (PX459) V2.0 (Addgene plasmid #62988) and lentiCRISPR v2 (Addgene plasmid #52961) were gifts from F. Zhang [76,77]. Plasmids of Dual Luciferase Reporter Assay (ISRE-Luc and Renilla (pRL-TK) plasmids), were gifts from D. Jin [78]. The plasmid of IRF3/5D, which is a constitutively active form of IRF3, was a gift from D. Jin [79]. Plasmids encoding shRNA against RanBP2 (shRNA3: TRCN0000003454, Sigma) were purchased from Sigma, St. Louis, MO, USA.

### 4.3. CRISPR-Cas9-Mediated Genome Editing

CRISPR-Cas9-mediated genome editing of RanBP2 in U2OS, HEK293, or Vero cells was performed as previously described [22]. Briefly, gRNA-dE3-1# (5′-GGGCTTTCTGCTCAGCGGT-3′), or gRNA-dE3-3# (5′-TGTAGCAGAAGAAAGTTGG-3′) targeting exon 21 of RanBP2 was inserted into pSpCas9(BB)-2A-Puro (PX459) V2.0. The PX459 V2.0 plasmids containing gRNA-dE3-1# and gRNA-dE3-3# were transfected into U2OS, HEK293 and Vero cells, respectively. After 48 h of transfection, cells were selected using 2 μg/mL puromycin. After about 2–4 weeks, single colonies were picked, grown, and subjected to Western blotting analysis of protein levels of RanBP2 and post-translational modification status of RanGAP1. Editing was verified by isolating genomic DNA, amplifying exon 21 by PCR and sequencing as previously described [22].

### 4.4. In Vivo Sumoylation Assay

In vivo STAT1 sumoylation was analyzed in unmodified and RanBP2-dE3 U2OS cells or unmodified and RanBP2-dE3-1 HEK293 cells as previously described [22]. Briefly, cells in 10 cm dishes were transfected with plasmids (2.5 μg each) expressing Flag tagged STAT1 (STAT1-Flag) or a lysine 703 to arginine mutant STAT1 (STAT1^K703R^-Flag) together with His-tagged SUMO2 or control vector using JetPRIME reagent (Polyplus) according to the manufacturer’s instructions. Twenty-four hours after transfection, the cells were harvested, and 10% were lysed in 2× SDS loading buffer (60 mM Tris-HCl pH 6.8, 1% SDS, 100 mM DTT, 5% glycerol) to generate an input sample. Ninety % of the cells were resuspended in 0.2 mL lysis buffer containing 6 M Guanidinium-HCl, 100 mM K_2_HPO_4_, 20 mM Tris-HCl (pH 8.0), 100 mM NaCl, 0.1% Triton X-100, and 10 mM Imidazole, and incubated on ice for 20 min. Lysates were passed through a 30 G needle five times. Purification of the His6-SUMO2 conjugates was performed on 50 μL of Ni^2+^-NTA agarose beads (Qiagen, Hilden, Germany) prewashed with lysis buffer and incubated for 2-3 h at room temperature with end-over-end rotation. The beads were washed once with 1 mL of lysis buffer, and three times with 1 mL of wash buffer containing 8 M urea, 0.1 M Na_2_HPO_4_/NaH_2_PO_4_ (pH 6.4), 0.01 M Tris-HCl (pH 6.4), 10 mM imidazole, 10 mM β-mercaptoethanol, and 0.1% Triton X-100 before elution in 2× SDS loading buffer.

### 4.5. Immunoblotting and Immunoprecipitation

These experiments were performed essentially as described previously with minor modifications [22]. In brief, when STAT1 phosphorylation was determined, cells were lysed with lysis buffer containing inhibitors for both protease and phosphatases (AbMole), and membranes were then blocked with 5% BSA prior to probing with antibodies. For STAT1 sumoylation analysis by immunoprecipitation followed by immunoblotting, cells were lysed with lysis buffer containing both protease inhibitor mixture cocktail (AbMole) and N-ethylmaleimide (10 mM, Sigma Aldrich). The antibodies with the indicated dilutions were as follows: His (mouse monoclonal, 1:1000 dilution, Abcam, Cambridge, UK, ab18184), RanBP2 (rabbit polyclonal, 1:1000 dilution, Abcam, ab112061, or mouse monoclonal, 1:200 dilution, Santa Cruz Co., Santa Cruz, CA, USA, SC-74518), tubulin (mouse monoclonal, 1:10,000 dilution, Abmart, Shanghai, China, M20023), Ubc9 (rabbit polyclonal, 1:1000 dilution, Cell Signaling, Danvers, MA, USA, 4918S), Flag (mouse monoclonal, 1:10000 dilution, Abmart, M20008M), GAPDH (rabbit polyclonal, 1:1000 dilution, ABGENT, San Diego, CA, USA, AP7873a), SUMO2 (rabbit polyclonal, 1:1000 dilution, Abcam, ab233222), STAT1 (rabbit monoclonal, 1:1000 dilution, Cell Signaling, 14994S), Phospho-STAT1(Tyr701) (rabbit monoclonal, 1:1000 dilution, Cell Signaling, 7649S), RanGAP1 (mouse monoclonal, 1:1000 dilution, Santa Cruz, sc-28322), actin (mouse monoclonal, 1:5000, Abmart, M20011S), JAK1 (mouse monoclonal, 1:1000 dilution, Cell Signaling, 50996S), histone H3 (rabbit polyclonal, 1:1000 dilution, ABclonal, Woburn, MA, USA, A2348). The relevant horse radish peroxidase (HRP) conjugated goat anti-rabbit (1:5000 dilution, Abmart, M21002), goat anti-mouse (1:5000 dilution, Abmart, M21001).

### 4.6. Dual Luciferase Reporter Assay

Unmodified (Ctrl) and RanBP2-dE3-1 cells were transfected with ISRE-Luc (Firefly luciferase ISRE reporter) plasmid (200 ng) and pRL-TK (Renilla luciferase) plasmid (internal control) (50 ng) using standard calcium phosphate precipitation. If co-transfected with IRF3/5D expression plasmid, cells were lysed by passive lysis buffer (Vazyme, Nanjing, China, DL-101-01) for the following dual luciferase assays at 24 h after transfection. In other cases, transfected cells were cultured for 24 h, and then mock treated (Mock) or treated with exogenous IFN-α (1000 U/mL) for another 8 h or 16 h. The luciferase activity was detected with a Dual Luciferase Reporter Assay Kit (Vazyme DL-101-01) according to the manufacturer’s instructions. The relative luciferase activity was obtained by normalizing the activity of firefly to that of Renilla luciferase, and then the ratio was normalized to the Ctrl cell line without IRF3/5D or IFN-α stimulation.

### 4.7. RNA Isolation and Quantitative Real-Time PCR

Total RNA from different cultured cells was isolated using TRIzol reagent (Invitrogen) according to the manufacturer’s protocol. The RNA was reversed transcribed into cDNA with a kit of StarScript II First-strand cDNA Synthesis Mix With gDNA Remover (GENESTAR, Houston, TX, USA, A224-10), and analyzed by quantitative real-time PCR (RT-qPCR) using Taq Pro Universal SYBR qPCR Master Mix, (Vazyme, Q712-02) as per manufacture’s protocol. RT-qPCR data was analyzed using the ΔCT method where the relative mRNA levels of the target genes were normalized to GAPDH (glyceraldehyde-3-phosphate dehydrogenase) expression. Primers used for RT-qPCR were, *Isg15* forward: 5′-GCCCACAGCCATGGGC-3′, reverse: 5′-TGTCCTGCAGCGCCACAC-3′; *Isg54* forward: 5′-ACTGCAACCATGAGTGAG-3′, reverse: 5′-CCTTTGAGGTGCTTTAGATAG-3′; *Isg56* forward: 5′-TACAGCAACCATGAGTACAA-3′, reverse: 5′-TCAGGTGTTTCACATAGGC-3′; and *GAPDH* forward: 5′-GTGGAAGGACTCATGACCAC-3′, reverse: 5′-CCTGCTTCACCACCTTCTTG-3′.

### 4.8. Cell Fractionation Assays

The cell fractionation assay was performed as previously described with minor modifications [22]. In brief, unmodified and RanBP2-dE3-1 HEK293 cells treated with IFN-α were washed with PBS followed by resuspension in Phi buffer containing 20 mM HEPES-KOH (pH 7.4), 150 mM potassium acetate, 5 mM magnesium acetate, complete protease inhibitor cocktail (AbMole), 10 mM PMSF and phosphatase inhibitor (AbMole). Cells were lysed in Phi buffer with 0.5% Triton X-100 for 1 min and spun at 1000× *g* for 5 min at 4 °C. The supernatant was kept as the cytoplasmic fraction and was spun at 15,000× *g* for 15 min to clear. Pelleted nuclei were washed two times to remove any unlysed cells. Tubulin and histone H3 were used as a cytosolic and nuclear loading controls, respectively.

### 4.9. Immunofluorescence Microscopy

Unmodified, RanBP2-dE3-1 or RanBP2-dE3-8 HEK293 cells, with or without Flag-STAT1-overexpression, were cultured on coverslips in 6-well plates and stimulated by IFN-α or IFN-β (1000 U/mL) for 30 min. Cells were washed three times with PBS and fixed with 4% paraformaldehyde in PBS for 15 min, followed by permeabilization with 0.1% Triton X-100 in PBS for 15 min at room temperature. Cells were further washed with PBS three times and blocked with 4% BSA in PBS for 1 h at room temperature. The coverslips were incubated with a primary antibody (Rabbit anti-STAT1, 1:500 dilution, Cell Signaling; or Rabbit anti-Flag, 1:800 dilution, Yeasen, Shanghai, China) in 1% BSA for 1 h at room temperature or overnight at 4 °C followed by three PBS washes. The cells were further stained with a secondary antibody (Alexa Fluor 594-conjugated goat anti-rabbit IgG, 1:700 dilution, Abcam). Samples were mounted in ProLong Gold Antifade Mountant with 4′,6-diamidino2-phenylindole (DAPI) (Invitrogen). Images were acquired on a ZEISS Axio Imager D2 fluorescence microscope. The nuclear/cytoplasmic fluorescence intensity of line scan graphs was measured from raw, unprocessed images using ImageJ software (version 1.51, Wayne Rasband, National Institutes of Health, Bethesda, MD, USA). Images shown in figures were adjusted for brightness and contrast using ImageJ software (version 1.51).

### 4.10. Virus Infection and Quantification

Herpes simplex virus type 1 tagged with green fluorescent protein (HSV-1-GFP) was a gift from Dr. Min-hua Luo [80,81]. Vesicular stomatitis virus tagged with green fluorescent protein (VSV-GFP) was a gift from Dr. George Fu Gao [82]. HSV-1-GFP and VSV-GFP were prepared by propagation in Vero cells. Virus titers were determined by standard plaque assay by using Vero cells. Prior to infection with viruses, unmodified (Ctrl) and RanBP2-dE3-1 HEK293 cells were pretreated with 1000 U/mL IFN-α or with medium only (Mock) for 8 h, and then infected with HSV-1-GFP or VSV-GFP at a multiplicity of infection (MOI) of 0.2 by diluting viruses in the serum-free medium for virus entry. Two hours later, the inoculum was removed, and cells were washed twice by pre-warmed 1×PBS followed by feeding with complete culture medium for 14 h. Then, the infected cells were observed for GFP expression as an indication of HSV-1 or VSV infection. Viral infections were analyzed by fluorescence microscopy and flow cytometry.

### 4.11. Flow Cytometry

Cells that were infected with HSV-1-GFP or VSV-GFP (MOI = 0.2) were harvested with cold 1× PBS followed by washing with PBS three times. After washing, the cells were subjected to analysis using the BD Accuri C6. FACS data were analyzed using FlowJo V10 software (version 10.8.1, Becton, Dickinson & Company, Ashland, OR, USA).

### 4.12. Statistical Analysis

The two-tailed Student’s *t*-test was used for all statistical analyses to calculate *p*-values. Differences between groups were considered statistically significant with *p*-value < 0.05. Results of statistical analysis are included in the corresponding figure legends.

## Data Availability

Data are contained within the article.

## Supplementary Materials

The supporting information can be downloaded at: https://www.mdpi.com/article/10.3390/ijms25010299/s1. References [83,84] are cited in the supplementary materials.
