# Supplementary material for: RanBP2/Nup358 Mediates Sumoylation of STAT1 and Antagonizes Interferon-α-Mediated Antiviral Innate Immunity
Source: Int J Mol Sci. 2023 Dec 25;25(1):299. doi: 10.3390/ijms25010299 (PMC10778711; doi:10.3390/ijms25010299)
Supplement: Supplementary file 1 [file ijms-25-00299-s001.zip › ijms-2748573-supplementary.pdf]

Supporting Information

RanBP2/Nup358 Mediates Sumoylation of STAT1 and Antagonizes Interferon-α-Mediated Antiviral Innate Immunity

Jiawei Li [1,†], Lili Su [1,†], Jing Jiang [1], Yifan E. Wang [2], Yingying Ling [1], Yi Qiu [2], Huahui Yu [1], Yucong Huang [1], Jiangmin Wu [1], Shan Jiang [1], Tao Zhang [1], Alexander F. Palazzo [2,*] and Qingtang Shen [1,*]

[1] Department of Immunology, School of Basic Medical Sciences, Fujian Medical University, Fuzhou 350108, China; lijiawei_69@163.com (J.L.); su993753394@163.com (L.S.); jiangjing601210@163.com (J.J.); lyy1650820@outlook.com (Y.L.); yuhuahui@fjmu.edu.cn (H.Y.); ychuang2000@163.com (Y.H.); fjmu379664877@outlook.com (J.W.); zy-if-72900yq@outlook.com (S.J.); zjdrzht@fjmu.edu.cn (T.Z.)

[2] Department of Biochemistry, University of Toronto, Toronto, ON M5G 1M1, Canada; yifa.wang@mail.utoronto.ca (Y.E.W.); tina.qiu@mail.utoronto.ca (Y.Q.)

* Correspondence: alex.palazzo@utoronto.ca (A.F.P.); qtshen1983@fjmu.edu.cn (Q.S.)

† These authors contributed equally to this work.

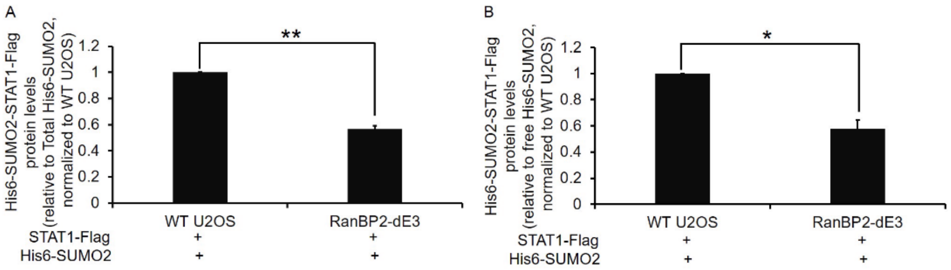

Supplemental data, Figure S1. RanBP2 mediates STAT1 sumoylation in cells. The isolated His6-SUMO2-STAT1-Flag, total His6-SUMO2 and free His6-SUMO2 protein levels were quantified using densitometry analysis and the ratio of His6-SUMO2-STAT1-Flag/ total His6-SUMO2 (A) or His6-SUMO2-STAT1-Flag/free His6-SUMO2 (B) was normalized to WT U2OS cells and plotted, with each bar representing the average of two independent experiments ± SEM. *P<0.05，**P<0.01 (Student's t-test).

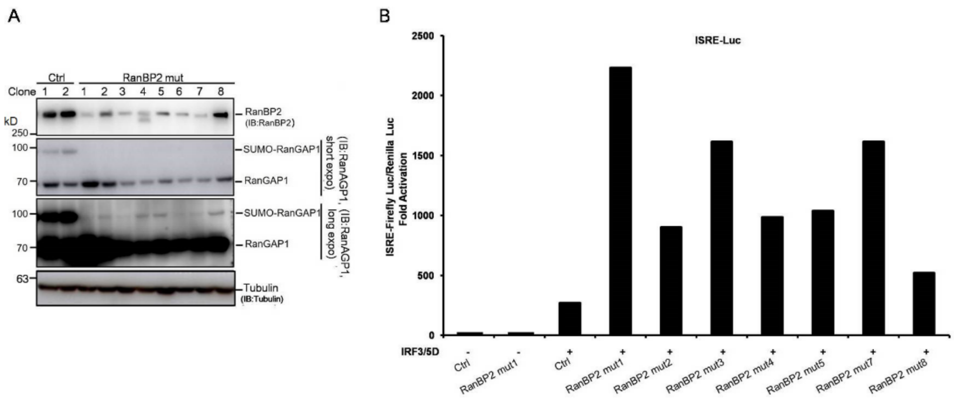

Supplemental data, Figure S2. RanBP2-dependent sumoylation inhibits the type I interferon-mediated signaling pathway in multiple gene-edited cell lines. (A) Immunoblotting analysis of RanBP2, RanGAP1, and Tubulin (loading control) in the whole-cell lysates of control (Ctrl1 and Ctrl2) and edited single cell clonies (RanBP2 mut1-8) generated by CRISPR/Cas9. (B) Dual-Luciferase Reporter (DLR) analysis of the effect of RanBP2 on the activation of interferon-stimulated response elements (ISRE) promoter induced by IRF3/5D, the active form of IRF3, consisting of 5-amino-acid residues mutation in the C-terminal domain of IRF3 which leads to its nuclear translocation and activates IFN-β promoter directly. Firefly and Renilla luciferase luminescence were measured and the ratio was normalized to the Ctrl cell line without IRF3/5D stimulation.

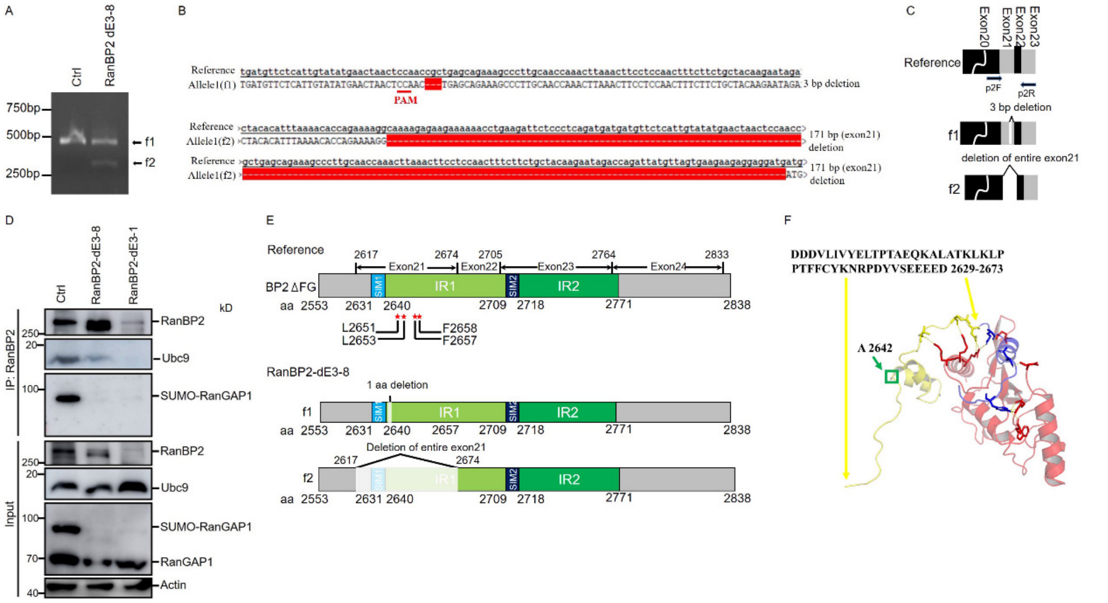

Supplemental data, Figure S3. Characterization of the RanBP2-dE3-8 cell line. (A) Genomic DNA amplification by PCR using p2F and p2R primers indicated in (C), of RanBP2 mRNA fragments in gRNA-targeted regions in unmodified HEK293 control cells (Ctrl) and RanBP2-dE3-8 cells. (B) PCR products of the two alleles (f1 and f2) from mutant RanBP2-dE3-8 HEK293 cells in (A) were sequenced and compared to exon 21 of the unmodified human RanBP2 gene (Reference). Note the PAM sites for gRNA（the guide RNA Protospacer Adjacent Motif）are indicated in red. (C) Schematic of the modified regions in the RanBP2 mRNAs. Also indicated are the PCR primers “p2F”and “p2R” for cDNA amplification, and the regions deleted in mRNAs produced from each RanBP2 allele (f1 or f2) in the RanBP2-dE3-8 cell line. (D) Various HEK293 cell lines were harvested and subjected to Co-IP assays. Cell lysates were separately immunoprecipitated with anti-RanBP2 antibody. Samples before (input) and after (IP) immunopurification were analyzed by immunoblotting using anti-RanBP2, anti-RanGAP1, anti-Ubc9 or anti-Actin antibody, respectively. (E) Schematic of mutant RanBP2 proteins from RanBP2-dE3-8 cells. BP2△FG: schematic of the SUMO E3 ligase domain encoded by a portion of the human *RanBP2* gene. SIM: SUMO interacting motif, IR: internal repeat. The amino acids that are denoted by asterisks have been shown to be required for SUMO E3-ligase activity [1]. (F) Crystal structure of the RanBP2 IR1 domain bound to Ubc9 from Protein Data Bank (PDB) (https://www.rcsb.org/). The IR1 domain of RanBP2 is indicated in blue, and Ubc9 is indicated in red. The partial deletion fragment (residues 2629-2673) of copy f2 of RanBP2-dE3-8 resides in the yellow-colored tail segments of RanBP2 IR1 domain. The position of Alanine 2642 that is deleted in copy f1 of RanBP2-dE3-8 is indicated by

the green box. The polar contacts between RanBP2 IR1 domain and Ubc9 are indicated in yellow dashed lines. The structure is adapted from Gareau et al. [2].

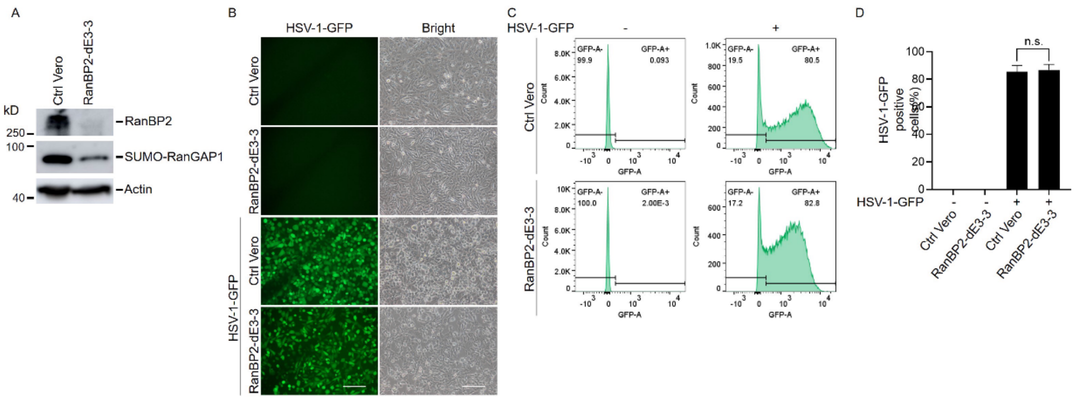

Supplemental data, Figure S4. Effect of RanBP2 mutant on viral infection in Vero cell lines. (A) Western blotting analysis of RanBP2, SUMO-RanGAP1 and Actin (loading control) in the whole-cell lysates of control (Ctrl Vero) and edited Vero cells by CRISPR/Cas9 system (RanBP2-dE3-3). (B) Ctrl Vero and RanBP2-dE3-3 cells were infected with HSV-1-GFP at an MOI of 0.5 for 8 h. The infected cells were observed for GFP expression as an indication of HSV-1 infection. Viral infection was analyzed by fluorescence microscopy (B) and flow cytometry (C-D). Representative images of virus infected (GFP-positive) cells were obtained by epifluorescence microscopy. Scale bar =400 μm (B). The percentage of virus infected (GFP-positive) cells in (B) were quantitated by flow cytometry (C) and plotted (D) with each bar representing the average of three independent experiments ± SEM. n.s. indicates no significant difference (Student's t-test).

References

1. Pichler, A.; Gast, A.; Seeler, J.S.; Dejean, A.; Melchior, F. The Nucleoporin RanBP2 Has SUMO1 E3 Ligase Activity. *Cell* **2002**, *108*, 109–120, doi:10.1016/S0092-8674(01)00633-X.
2. Gareau, J.R.; Reverter, D.; Lima, C.D. Determinants of Small Ubiquitin-like Modifier 1 (SUMO1) Protein Specificity, E3 Ligase, and SUMO-RanGAP1 Binding Activities of Nucleoporin RanBP2. *Journal of Biological Chemistry* **2012**, *287*, 4740–4751, doi:10.1074/jbc.M111.321141.
